# Preparation and Characterization of Coated ZnFe_2_O_4_ Magnetic Nanoparticles for Potential Use as Magnetic Particle Imaging Tracers

**DOI:** 10.1021/acsomega.5c03598

**Published:** 2026-01-12

**Authors:** Gulsum Caliskan, Sevil Ozer, Muhammad Irfan, Nurcan Dogan

**Affiliations:** † Department of Physics, 52962 Gebze Technical University, 41400 Kocaeli, Turkey; ‡ 209002 TUBITAK National Metrology Institute (UME), 41400 Kocaeli, Turkey; § Department of Electronics Engineering, Gebze Technical University, 41400 Kocaeli, Turkey; ∥ Department of Physics Engineering, Istanbul Technical University, 34469 Istanbul, Turkey

## Abstract

Magnetic nanoparticles (MNPs) exhibit unique behaviors that make them appealing for a variety of clinical applications, including their use as tracer agents in magnetic particle imaging (MPI) and magnetic resonance imaging (MRI) systems, as heat generators in hyperthermia treatment, and in targeted drug delivery. We prepared maghemite cores (ZnFe_2_O_4_) by coprecipitation of Zn­(II) and Fe­(III) salts with ammonium hydroxide, followed by a mixture under N_2_ gas. The cores were coated with coating agents: citric acid monohydrate (CA), l-ascorbic acid (AA), and l-(+)-tartaric acid (TA). State-of-the-art techniques characterized their structural and magnetic properties. XRD results showed that all the particles were consistent with the crystal structure of ZnFe_2_O_4_, and the average crystallite size ranged from 14 to 19 nm. The hydrodynamic diameters were found between 112 and 218 nm. Zeta potential values for negatively charged particles in the range of −29.7 to −60.3 mV provided good colloidal stabilization. FTIR analysis showed the existence of coating agents on the structures. SEM measurements confirmed the spherical geometry of all samples. All synthesized ZnFe_2_O_4_ nanoparticles showed a superparamagnetic behavior. The primary aim of this study was to investigate the potential ability of coated ZnFe_2_O_4_ nanoparticles to serve as molecular magnetic contrast agents in imaging studies for MPI systems. Consequently, the prepared samples were also tested with magnetic particle spectroscopy (MPS), and the results were compared with those of commercial reference tracers, Perimag and VivoTrax, which are used in MPI. The MPS results show that ZnFe_2_O_4_ samples yielded the best results, with the shortest effective relaxation time (2.09 μs for ZnFe_2_O_4_@CA and 2.85 μs for ZnFe_2_O_4_@AA) and an excellent spatial resolution (fwhm, 5.89 mT for ZnFe_2_O_4_@CA and 5.14 mT for ZnFe_2_O_4_@AA). The structural and magnetic characterizations of CA, AA, and TA-coated ZnFe_2_O_4_ nanoparticles indicate that they are suitable for biomedical applications and especially have great potential as tracer agents for MPI.

## Graphical abstract

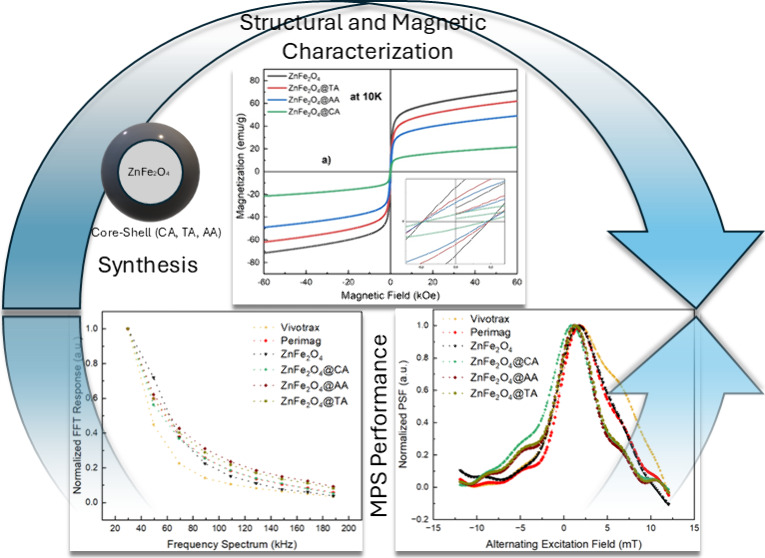

## 1 Introduction

Magnetic particle spectroscopy (MPS), derived from magnetic particle imaging (MPI) technology, is a promising and versatile analytical technique that utilizes the nonlinear magnetic response of superparamagnetic iron oxide nanoparticles (SPIONs).^1–6^ The physical behaviors of the SPIONs at the nanoscale influence performance in magnetic particle imaging, which employs alternating magnetic fields and a combination of magnetic field gradients to detect changes in nanoparticle magnetization and depict their spatial distribution.^7–10^ MPS has evolved into a significant instrument for accurately detecting SPIONs within biological environments.^11–13^ SPIONs hold considerable potential in clinical applications, such as delivering drugs or therapeutic agents, treating cancer through hyperthermia, and serving as contrast agents in diagnostic imaging.^10,14–18^ The superparamagnetic nature of SPIONs guarantees their absence of residual magnetization when an external magnetic field is not present, thereby aiding in the prevention of agglomeration in vivo. In conjunction with their biocompatibility and stability, this attribute positions SPIONs as highly suitable candidates for various potential applications.

Spinel ferrites with the general formula MFe_2_O_4_ (M = Zn^2+^, Ni^2+^, and Mn^2+^) are extensively studied materials that attract growing interest due to their unique properties, such as low coercivity and controlled magnetization. Zinc ferrite (ZnFe_2_O_4_) is particularly noteworthy among spinel ferrites because of its exceptional properties, including chemical stability, low toxicity, and the sensitivity of thermal and magnetic behaviors to nanoparticle size.^19,20^ Zinc ferrite nanoparticles can be synthesized using various methods, including sol–gel,^21^ hydrothermal,^22^ combustion,^23^ coprecipitation,^24^ thermal decomposition,^25^ and microemulsion.^26^ Among these techniques, hydrothermal and coprecipitation processes are straightforward, cost-effective, and use nontoxic raw materials. These methods offer several advantages: they operate at low temperatures, yield small particle sizes, exhibit high porosity, allow rapid preparation, maintain high purity, ensure solid chemical homogeneity, demonstrate crystallinity, and feature a simple process.^27–30^

The effective application of nanoparticles depends on their colloidal stability within an aqueous medium and on the meticulous regulation of their shape, size, and size distribution, all of which are critical in determining the chemical and physical properties of the nanocomposite. Naked nanoparticles (NPs) have high surface energy and aggregate to minimize total surface energy. To prevent the aggregation of magnetic nanoparticles due to attractive van der Waals forces, magnetic dipole–dipole interactions, high surface energy, and electrostatic interactions from insufficient surface charge stabilization, the particles must be coated with various complex agents that enhance stability and nontoxicity for biomedical applications.^31,32^ Different biocompatible synthetic and natural polyacids, such as dimercaptosuccinic acid, tartaric acid, citric acid, glutamic acid, and aspartic acid, have been utilized as capping agents and reported in the literature.^33–35^

This study demonstrates that surface modification of ZnFe_2_O_4_ with polyacids effectively enhances its stability and guarantees strong performance as tracers in molecular imaging. The manufacturing and surface modification processes were conducted in an aqueous medium, free of harmful chemicals. Employing a combination of hydrothermal and coprecipitation methods, we synthesized ZnFe_2_O_4_ samples coated with citric acid (CA), ascorbic acid (AA), and tartaric acid (TA). We studied them thoroughly for changes in colloidal stability and structural and magnetic properties using various state-of-the-art techniques. The selection of CA, AA, and TA as coating agents for zinc ferrite (ZnFe_2_O_4_) nanoparticles in MPI and MPS applications is based on their ability to enhance colloidal stability, unique magnetic response, dispersibility, and biocompatibility. CA improves dispersibility, stabilizes surface charge, and prevents aggregation, which is a key factor in selecting it as a capping agent;^36^ AA provides antioxidant protection,^37^ and TA ensures strong surface binding.^38^ These coating agents exhibit significant behavior, making them promising candidates for MPS and MPI applications. To determine the MPI performance of the ZnFe_2_O_4_ samples, we used magnetic particle spectroscopy (MPS).^39,40^ MPS measures the nonlinear dynamic magnetic response of MNPs when exposed to an alternating magnetic field. While certain MPS systems utilize zero-dimensional excitation, more advanced MPS methods can leverage multidimensional magnetic field excitations to better characterize nanoparticle behaviors.^41,42^ MPS assesses the nonlinear dynamic magnetic response of magnetic nanoparticles (MNPs) when exposed to an alternating magnetic field, offering insights into their applicability for MPI. Although MPS does not provide spatial details like MPI does, it continues to be an effective tool for evaluating the magnetic properties of MNPs in conditions pertinent to MPI. Furthermore, relaxivity measurements were carried out to evaluate the capability of the coated ZnFe_2_O_4_ samples as contrast agents in imaging systems. MPS performance was compared to our previous results^43^ from the two commercial MNP systems, Perimag (Micromod Partikeltechnologie GmbH) and VivoTrax (Magnetic Insight, Inc.), for comparison. In addition to confirming the superparamagnetic behavior and excellent tracer performance of ZnFe_2_O_4_ nanoparticles, this study highlights an important insight: surface chemistry is as crucial as particle size in determining MPI performance. Our findings show that AA- and TA-coated ZnFe_2_O_4_ nanoparticles can achieve relaxation times and spatial resolutions that surpass commercial standards. The outstanding performance of ZnFe_2_O_4_@AA, in particular, can be attributed to the antioxidant properties of ascorbic acid, which helps maintain core magnetic dynamics by preventing surface oxidation and spin disorder. This result emphasizes the novelty of our approach, demonstrating that systematically varying coating ligands under controlled synthesis conditions allows for the design of next-generation MPI tracers, where tailored surface chemistry improves both colloidal stability and magnetic relaxation behavior.

## 2 Methods

### 2.1 Materials

In the preparation process, the following pure chemicals were used: ferric chloride hexahydrate (FeCl_3_.6H_2_O) and zinc chloride (ZnCl_2_) salts, and ammonium hydroxide (NH_4_OH, with 25% of ammonia in water) with a minimum purity of 99% provided by Sigma-Aldrich. To prepare the coated ZnFe_2_O_4_ nanoparticles, citric acid monohydrate (CA), l-ascorbic acid (AA), and l-(+) tartaric acid (TA), also from Sigma-Aldrich, with 99.9% purity, were used.

### 2.2 Synthesis of ZnFe_2_O_4_ Nanoparticles

The two-step model, including coprecipitation and hydrothermal methods,^43,44^ was used to synthesize the coated ZnFe_2_O_4_ core/shell structure. Aqueous solutions were prepared by dissolving 4 mmol of FeCl_3_·6H_2_O and 2 mmol of ZnCl_2_ salts in 40 mL of DI water. The solution was stirred with a mechanical stirrer at 500 rpm at room temperature (25 °C) for 2 min while nitrogen gas was supplied. Next, 5 mL of NH_4_OH was added to the solution. This solution was transferred to a Teflon-lined autoclave, which was sealed and maintained at 180 °C for 12 h in the furnace before being cooled to room temperature. The precipitates underwent three washes with DI water before being filtered using a permanent magnet. The final precipitate was dissolved in 50 mL of DI water containing 3 mmol of coating agents (CA, AA, and TA) using an ultrasonic bath at 65 °C. Then, 1 mL of NH_4_OH was introduced to the final solution. The mixture was mechanically stirred for 30 min under nitrogen gas using the coprecipitation method to achieve the core/shell structure. The nitrogen gas flow was stabilized during this process to shield the nanoparticles from agglomeration effects caused by environmental conditions. The final products were rinsed 2–3 times with DI water. Then, the fine precipitates were collected using a magnet and dried at 60 °C for 2 hour in an oven before characterization. The synthesis process steps of the ZnFe_2_O_4_ NPs are shown in Figure 1.

**Figure 1:**
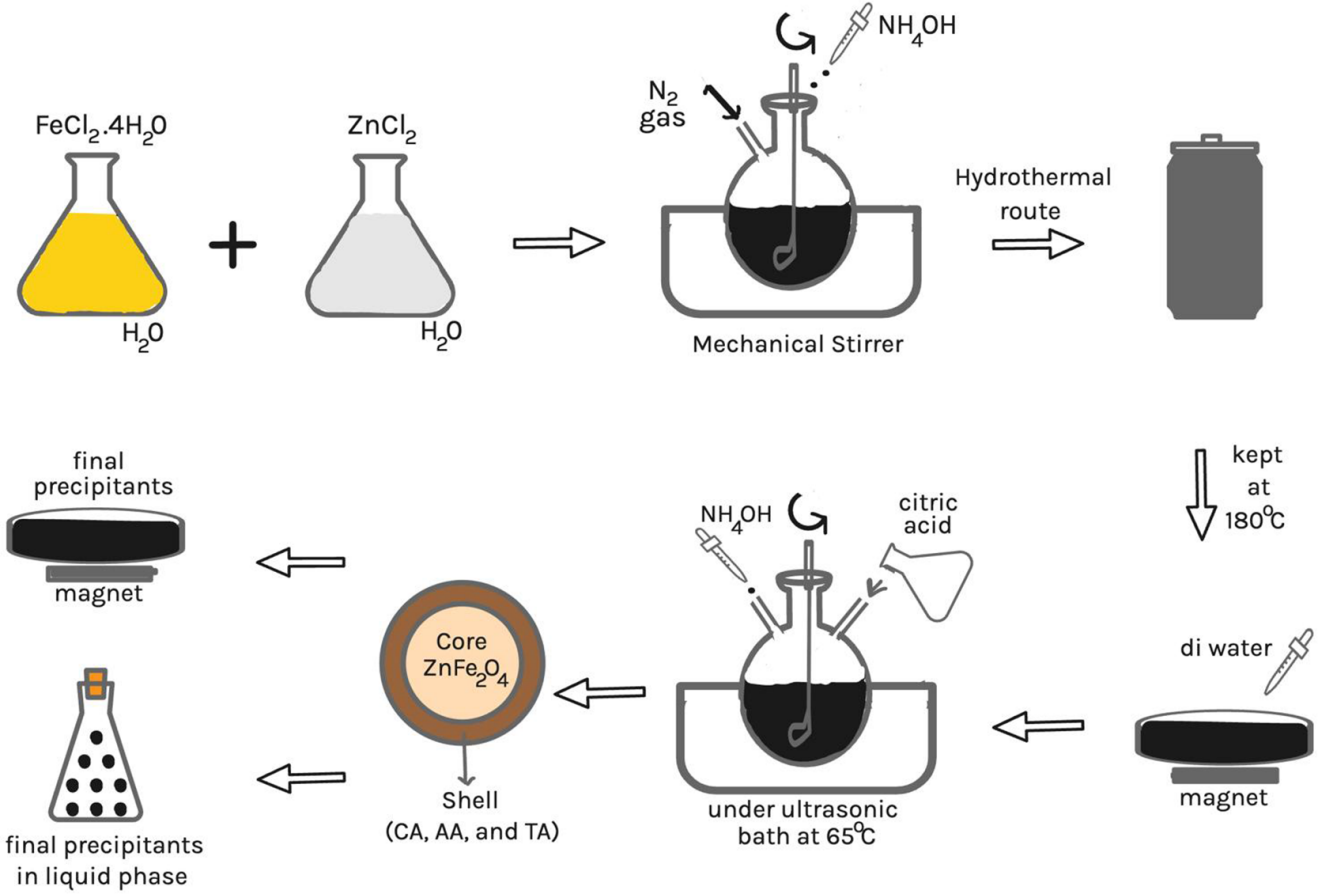
Schematic diagram illustrates the coprecipitation and hydrothermal procedures for synthesizing ZnFe_2_O_4_ nanoparticles.

### 2.3 Characterization of ZnFe_2_O_4_ NPs

Structural characterization of coated ZnFe_2_O_4_ NPs was performed using a powder X-ray diffractometer (XRD) with a Rigaku CuKα radiation source (Rigaku X-ray 2200 diffraction) over a 2θ range of 20° to 90°, with a scan step of 0.02° at 40 kV and 35 mA. The coating conditions were examined using PerkinElmer L160000R Fourier transform infrared (FTIR) spectroscopy in the 4000 to 600 cm^^–^1^ range. We analyzed the shape and microstructure of samples using a JEOL 6700 Scanning Electron Microscope (SEM) coupled with energy-dispersive X-ray spectroscopy (EDS). The size and zeta potential in the hydrodynamic state of coated ZnFe_2_O_4_ NPs were assessed using dynamic light scattering (DLS) techniques with the Zetasizer Nano-ZS90 System (Malvern Inc.). The thermal behavior of ZnFe_2_O_4_ NPs was studied using a thermogravimetric analyzer (TGA - PerkinElmer, Pyris Series STA-8000) over a temperature range of 30 to 800 °C, with a constant nitrogen flow and a heating rate of 20 °C/min. ESR measurements were conducted to investigate the spin dynamics of nanosized systems using a JEOL (Brand-JES-FA300) electron spin resonance (ESR) spectrometer alongside a Bruker EMX (X-band spectrometer). The ESR spectra were obtained at room temperature (296 K) in a DC magnetic field range of 0 to 1000 mT. All samples were housed in quartz ESR tubes, and the potential magnetic influences from the cavity and quartz tubes were subtracted from the measurements. The magnetic properties of the NPs were determined using the Physical Properties Measurement System (PPMS) (Quantum Design Model 6000) at temperatures of 10 K, 300 K, and 400 K. Finally, MPS characterization of coated ZnFe_2_O_4_ NPs was conducted with a homemade relaxometry system^39,40^ at a frequency of 9.9 kHz and a 15 mT sinusoidal excitation field.

## 3 Structural and Phase Characterization of Synthesized Nanomaterials: Results and Discussion

### 3.1 FTIR Results

FT-IR provides information about the presence of functional groups and the nature of chemical bonds in the samples. Figure 2 shows the FT-IR spectrum of the uncoated and CA, AA, and TA-coated ZnFe_2_O_4_ samples over the range of 4000–600 cm^–1^. All the coated samples displayed sharp peaks near 1604 cm^–1^, attributed to the C=O stretching, indicating the presence of free carbonyl groups in the coating samples. The peak around 1388 cm^–1^ is associated with the C–O bond in ascorbic acid, tartaric acid, and citric acid. These distinct peaks confirm the presence of coating materials on the synthesized nanoparticles.^43,45^ The broadband observed at 3312 cm^–1^ results from the stretching and bending vibrations of the H–O–H bond on the surface, indicating the presence of physiosorbed water molecules and organic moieties in all ZnFe_2_O_4_ samples.^33^ The observed bands at about 1050 cm^–1^ and 1100 cm^–1^, which are seen as stronger for ZnFe_2_O_4_@AA sample, can be ascribed to the stretching vibration of CH_2_ bonds and C=O groups, respectively.^46^ The sharp and strong intensity band at 752 cm^–1^ corresponds to the characteristic Fe–O magnetite band and Zn–O vibration. The two primary bonds verify the presence of metal ions, which are Fe^3+^ and Zn^2+^ ions at tetrahedral (A) or octahedral sites (B) and oxygen ions O^2–^, respectively.^47^ A weak yet persistent transmittance band appears just above 1250 cm^–1^ (not indicated in Figure 2) in bare and coated ZnFe_2_O_4_ samples. This suggests the vibration likely arises from the intrinsic structural properties of the spinel lattice or residual precursor materials from synthesis. Additionally, minor Zn/Fe–O–Zn/Fe bending vibrations, surface hydroxyl groups, or defect-related modes may contribute to this feature.^48^ There were no considerable differences in peak positions within the sample pattern. The FT-IR spectra confirmed the formation of a ferrite phase and the successful application of a coating agent on the nanoparticles’ surface.

**Figure 2:**
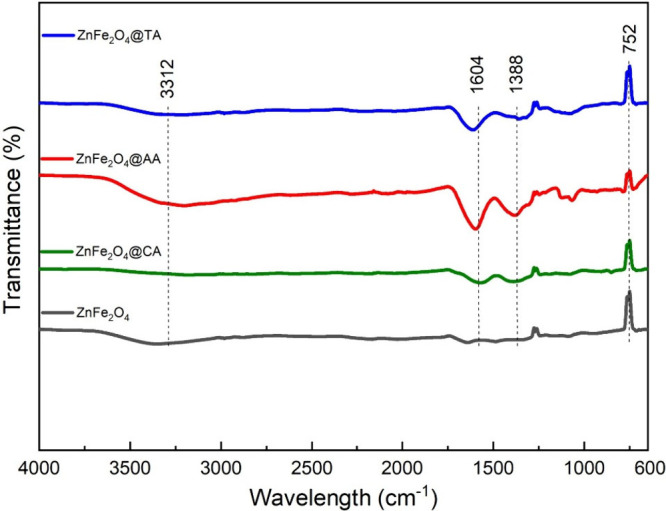
FT-IR spectrum of ZnFe_2_O_4_@TA, ZnFe_2_O_4_@AA, and ZnFe_2_O_4_@CA, and uncoated ZnFe_2_O_4_, nanoparticles.

### 3.2 XRD Results

XRD was used to assess the structural properties and phase composition of the coated and uncoated zinc ferrite nanoparticles, as illustrated in Figure 3. The diffraction peaks at (220), (311), (400) (422), (511), and (440) correspond to the face-centered cubic spinel structure of ZnFe_2_O_4_ nanoparticles, demonstrating excellent agreement with JCPDS card no. 22-1012.^46,47^ No further reflections were observed, confirming the starting material is phase pure. The XRD patterns of the AA and TA-coated samples align well with the JCPDS reference and the pristine ZnFe_2_O_4_ nanoparticles. This indicates that the ascorbic acid and tartaric acid coating process does not alter the structure. Only the CA-coated samples show impure and secondary phases of hematite. The observed formation of hematite in ZnFe_2_O_4_@CA nanoparticles arises from the combined effect of citric acid’s strong Fe^3+^ chelation and the high-energy environment of ultrasonic treatment, which promotes Fe^3+^ extraction and reprecipitation under localized oxidative conditions.^49^ Furthermore, Citric acid, a relatively strong organic acid, markedly lowers the pH of the reaction medium. This acidic environment enhances the solubility of Fe^3+^ ions and may facilitate their partial reduction to Fe^2+^, thereby promoting the formation of hematite (Fe_2_O_3_) as a secondary phase. Since the coating agents have minimal impact on the phase development of zinc ferrite, the XRD evaluation was performed solely for crystal phase analysis. The reference for spinel Fe_2_O_3_ is marked with a star in the figure.

**Figure 3:**
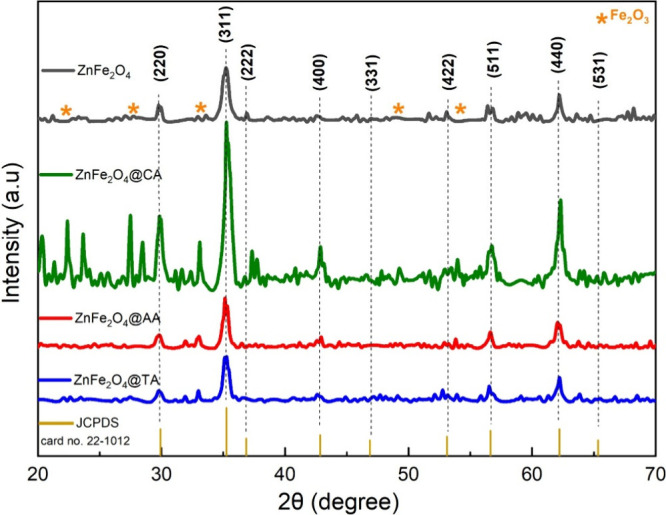
XRD patterns of uncoated ZnFe_2_O_4_, ZnFe_2_O_4_@CA, ZnFe_2_O_4_@AA, and ZnFe_2_O_4_@TA nanoparticles.

The main peak at (311) plane was utilized to estimate the average crystallite size through the Scherrer formula;^50^

1 $$D_{xrd}=\frac{K\lambda }{\beta \;\cos\theta }$$

where *D* is the average crystallite size, λ represents the wavelength of X-ray (1.5418 Å) used. *K* denotes the Scherrer constant (0.89), β indicates the full width at half-maximum intensity (fwhm) of the X-ray diffraction lines, and θ signifies the Bragg peak angle. The crystallite sizes of the samples were calculated to be in the range of 14–19 nm. The particle size of ZnFe_2_O_4_ nanoparticles is observed to increase with surfactants, except for the CA-coated sample, as shown in Table 1.

The average crystallite size is calculated using the highest peak in XRD data and typically reflects the size of a single crystalline region. However, nanoparticles may consist of many crystallites, so the nanoparticle size can be larger than the average crystallite size.

**Table 1:** XRD Data of the Zinc Ferrite (ZnFe_2_O_4_) Samples with Biocompatible Coatings

sample no.	*D*_xrd_ (nm)	*A* (Å)	ρ (g/cm^3^)	δ (10^15^ m^–2^)	*S* (10^4^ m^2^/g)	ε (10^*–*3^)
ZnFe_2_O_4_	14	8.41	5.3	4.5	7.5	7.6
ZnFe_2_O_4_@TA	16	8.40	5.4	3.6	6.6	6.7
ZnFe_2_O_4_@AA	19	8.40	5.4	2.7	5.8	6.0
ZnFe_2_O_4_@CA	14	8.44	5.3	4.7	7.7	7.8

To examine how the coating affects the nanoparticles’ structural characteristics, including the lattice parameter (*a*), dislocation density (*δ*), X-ray density (*ρ*), and specific surface area (*S*), the following equations from XRD data^50^ were used to calculate these parameters. The computed values are listed in Table 1.

The lattice parameter, *a*, for nanophased zinc ferrite, relevant to the observed reflections, was calculated by using eq 2:

2 $$a=d_{hkl}\sqrt{h^{2}+k^{2}+l^{2}}$$

where *h*, *k*, and *l* are the Miller Indices and *d*_*hkl*_ is *d*-spacing, indicating the distance between the atoms in the planes. The lattice parameter value of ZnFe_2_O_4_ samples was about 8.4 Å, consistent with the zinc ferrite samples’ lattice constant reported in the literature.^51,52^ X-ray density (ρ) values have been calculated using the following eq 3:

3 $$\rho =\frac{ZM}{Na^{3}}$$

where *M* indicates the sample’s molecular weight, and *N* denotes Avogadro’s number. The number of molecules in the spin–lattice’s unit cell is represented by *Z* = 8 for the spinel cubic structure. The density values of uncoated and coated ZnFe_2_O_4_ samples range from 5.3 to 5.4 g/cm^3^. Table 1 indicates that the AA and TA-coated zinc ferrite sample has a higher density than the CA-coated zinc ferrite sample.

Based on the average particle size values obtained from eq 1, the dislocation density value of the zinc ferrite samples can be determined using eq 4:

4 $$\delta =1/D^{2}$$

The dislocation density value reflects the degree of crystallinity in the nanoparticle profile. Results indicate an increased dislocation density value only when the CA is added. Smaller dislocation density values in ZnFe_2_O_4_ with AA and TA-coating agents suggest that these nanoparticles were produced with high crystallinity.

By using average crystallite size values obtained from eq 1 and density (ρ) values from eq 3, specific surface area (*S*) can be determined as follows, eq 5:

5 $$S=6\times 10^{3}/\rho D$$

The specific surface area decreases as the crystallite size increases. The substantial surface area of ZnFe_2_O_4_ significantly contributes to its high anisotropy, consistent with the findings from the PPMS analysis of the samples.

The strain-induced broadening (*ε*) in samples is due to crystal imperfection. It has been calculated to analyze the distortion:^53,54^

6 $$\varepsilon =\frac{\beta }{4\;\tan\theta }$$

where θ is the Bragg peak angle and β is the diffraction peak’s full width at half of its highest intensity (FWHM). The lattice strain increases as the crystallite size decreases and is strongly correlated with the crystallite size. The sample with AA coatings exhibited the lowest lattice strain value in conjunction with the highest crystallite size value. While all three coating agents interact with the surface of the nanoparticles, their effects on size vary. TA and AA facilitate moderate and substantial increases in size, respectively, whereas CA exhibits a negligible impact. These discrepancies emerge from the distinct chemical mechanisms employed by each agent to affect the nucleation and growth processes during nanoparticle synthesis, such as redox behavior and chelation strength.

### 3.3 SEM/EDX Results

SEM analysis was conducted to gain further insights into the morphology of the coated materials. The SEM images in Figure 4 indicate the uncoated and CA, AA, and TA-coated zinc ferrite nanoparticles. The uncoated sample data previously presented in reference^43^ has also been reintroduced in this study to enable a comparative analysis with coated samples using various agents. This inclusion facilitates a more thorough evaluation of the effects of surface modification on the material’s properties. The sample particles have a spherical shape with high agglomeration. Because most of the particles are agglomerated, the exact size of the particle using SEM cannot be determined. It is observed that some parts of ZnFe_2_O_4_@CA nanoparticles exhibit a plate structure and crucks, as shown in Figure 4b. The reason for this might be the high dry reaction temperature. SEM images also reveal how the particles have a high agglomeration tendency due to magnetic dipole–dipole interaction, the attractive van der Waals Forces, high surface energy, electrostatic interactions with insufficient surface charge stabilization and the large surface area associated with reduced size.^55,56^ The uncoated sample (Figure 4a), and coated samples with AA (Figure 4c), and TA (Figure 4d) have a relatively better round shape as compared with the CA-coated sample (Figure 4b). Theoretical and experimental reports have established that dipole–dipole interactions may lead to either the formation of chains or random agglomeration into spherical clusters, contingent upon the anisotropy, size, and concentration of the magnetic nanoparticles (MNPs). These interactions become particularly significant after drying, as solvent molecules no longer separate the particles. Although Zn doping may modify the magnetic properties to some extent, residual magnetization still facilitates nanoparticle clustering. In aqueous environments, surface charge can prevent particle aggregation through electrostatic repulsion. However, the surface charge may decrease or neutralize during drying, reducing repulsive forces and leading to particle clustering. Therefore, to mitigate nanoparticle agglomeration, the following strategies can be implemented: increasing the coating concentration enhances the functional group binding affinity to zinc-ferrite surfaces, and extending the coprecipitation duration may improve the uniformity of the protective layer.

**Figure 4:**
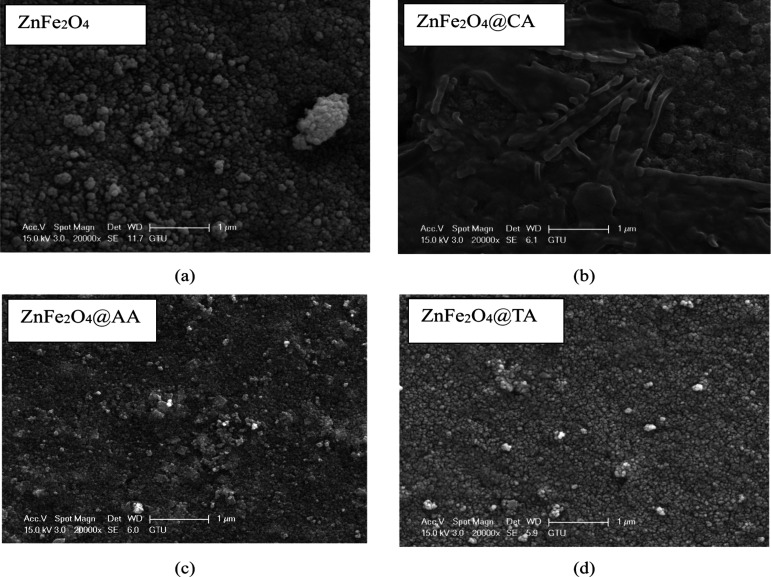
SEM pattern of (a) ZnFe_2_O_4_, coated: (b) ZnFe_2_O_4_@CA, (c) ZnFe_2_O_4_@AA, and (d) ZnFe_2_O_4_@TA nanostructures.

Moreover, the purity and elemental composition of the ZnFe_2_O_4_ nanostructures were analyzed through EDX, as illustrated in Figure 5. It is abundantly evident that the EDX spectrum confirmed the presence of the coating agents on the crystal of ZnFe_2_O_4_ samples. Table 2 lists the atomic and mass percent compositions.

**Figure 5:**
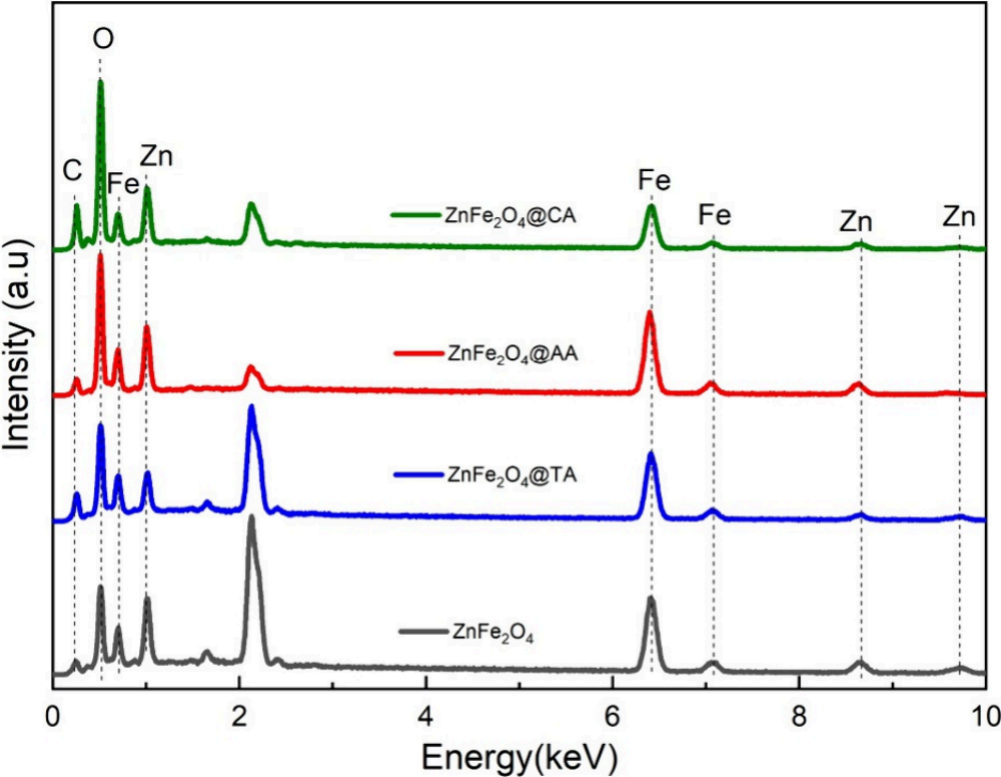
EDX spectra of ZnFe_2_O_4_@CA, ZnFe_2_O_4_@AA, ZnFe_2_O_4_@TA, and bare ZnFe_2_O_4_ nanoparticles.

**Table 2:** Elemental Composition of the Produced Zinc-Based MNPs

	element ratio (wt %)				
sample	Zn	Fe	O	C	total
ZnFe_2_O_4_	28	58	12	-	100
ZnFe_2_O_4_@TA	13	54	17	15	100
ZnFe_2_O_4_@AA	23	51	17	8	100
ZnFe_2_O_4_@CA	14	34	31	20	100

The spectrum displayed peaks corresponding to Zn, Fe, O, and C, with no other elements identified, which is consistent with XRD measurements. The elemental composition of Zn and Fe in the uncoated sample, as presented in Table 2, is relatively close to the theoretical value. The Zn/Fe ratio in uncoated ZnFe_2_O_4_ is approximately 0.48 (28% Zn, 58% Fe). However, for the coated samples, the ratios change: ZnFe_2_O_4_@TA has a Zn/Fe ratio of about 0.24 (13% Zn, 54% Fe), ZnFe_2_O_4_@CA is 0.41 (14% Zn, 34% Fe), and ZnFe_2_O_4_@AA is 0.45 (23% Zn, 51% Fe). These variations can be attributed to the surface effects of the organic coatings, which reduce the visibility of metal elements in the EDX signal, rather than reflecting true stoichiometric changes.

Additionally, carbon was detected only in the coated samples, with amounts ranging from approximately 8% to 20%, further confirming successful surface modification. Since all samples were synthesized under identical conditions with a Zn^2+^:Fe^3+^ precursor ratio of 1:2, we believe the core stoichiometry has been preserved. Therefore, the observed differences in elemental ratios are attributed to the surface effects of the coatings and the inherent limitations of the EDX technique.

### 3.4 Dynamic Light Scattering (DLS) and Zeta Potential (ZP) Results

The colloidal stability of the samples in the aqueous system has been assessed using dynamic light scattering (DLS). Figure 6a shows the distribution of the total particle sizes of NPs. The hydrodynamic size (*d*_nm_) of CA, AA, and TA-coated ZnFe_2_O_4_ samples ranged from 114.5 to 218 nm but decreased to about 112 nm for the uncoated ZnFe_2_O_4_ samples. Therefore, adding a coating agent shifts the size distribution toward a slightly larger value. The peaks between 10^3^ and 10^4^ in Figure 6 suggest the existence of aggregated nanoparticles in the solution.^57,58^ Consequently, the measured size appears larger than the actual particle core–shell size. This may also occur if the conditions, such as pH or ionic concentration of the solvent, favor aggregation.

**Figure 6:**
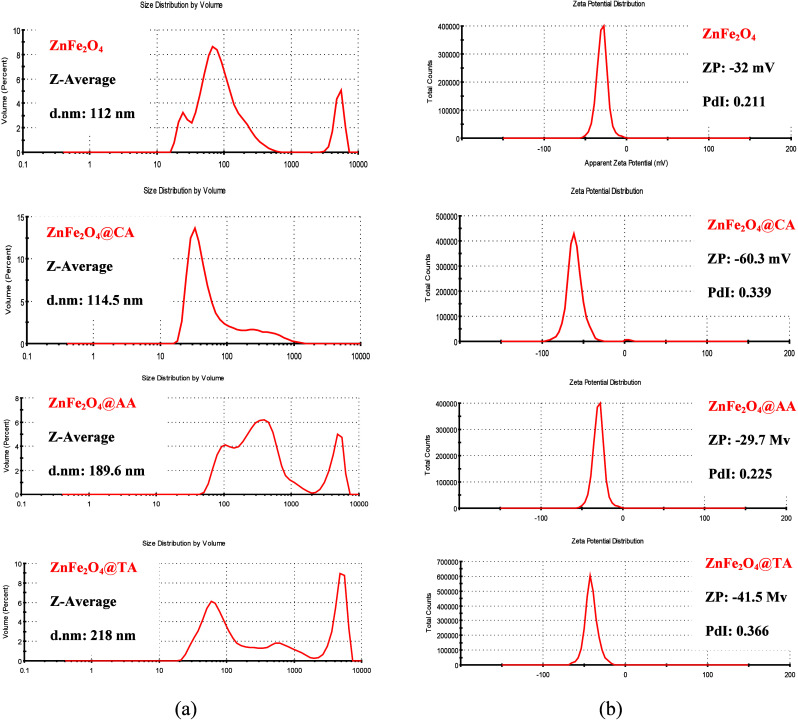
(a) DLS and (b) Zeta potential analyses of the bare ZnFe_2_O_4_, ZnFe_2_O_4_@CA, ZnFe_2_O_4_@AA, and ZnFe_2_O_4_@TA nanoparticles.

In DLS, it is common for particle sizes to be significantly larger than those measured by other methods such as SEM, TEM, or XRD; this difference arises from the inherent working principle. It is assumed that a hydration layer surrounds the particle, as the particles undergo Brownian motion in suspension. The hydrodynamic diameter is calculated from the translational diffusion coefficient, which depends not only on the particle’s core size but also on the medium’s viscosity, temperature, and the Boltzmann constant. Therefore, it is primarily influenced by factors such as the particle’s surface structure, shape, and fluid viscosity, resulting in a size that is usually larger than the actual nanoparticle size measured by other methods.^59^

Fluidic stability of uncoated and CA, AA, and TA-coated dispersed ZnFe_2_O_4_ samples in aqueous media was investigated by measuring the zeta potential, with Zeta potential (ZP) distributions displayed in Figure 6b. A key component of colloidal stability is the magnitude of the zeta potential, which for electrostatically stable colloidal suspensions typically shows values greater than +30 mV or less than −30 mV.^60,61^ Although the zeta potential serves as a valuable indicator of colloidal stability, particularly in relation to electrostatic repulsion; however, its application to nanoparticles necessitates meticulous contextualization. For instance, iron oxide nanoparticles (IONPs) that are coated with small organic acids frequently display zeta potential values ranging within ± 30 mV, yet they retain colloidal stability as a result of steric effects and specific surface interactions.^62,63^ Van der Waals interparticle forces will ultimately cause dispersions with low zeta potential to aggregate. Introducing ionizable functional groups on nanoparticles enables modulation of surface charge, which may be either positive or negative, depending on the nature of the solution.^64^ CA has several carboxylate groups that strongly adhere to the nanoparticle surface, creating a high density of negative charges and greatly improving electrostatic stability.^62^ TA, featuring two carboxyl and two hydroxyl groups, provides fewer ionizable groups compared to citric acid. The carboxyl groups enhance surface charge, while the hydroxyl groups can form hydrogen bonds, which may neutralize the surface charge or generate localized dipoles.^65^ Conversely, the AA-coated sample (−29.7 mV) showed a zeta potential comparable to the uncoated version, indicating a lesser electrostatic effect, probably due to AA’s limited dissociable acidic groups and the prevailing steric/antioxidant stabilization from its enediol structure. In this case, the zeta potential values for all samples ranged from −29.7 to −60.3 mV, confirming their good colloidal stability. Among all the coated samples, CA-coated zinc ferrite, with the highest charge value, may offer the best stability for the magnetic fluids. While a strongly negative zeta potential, such as −60 mV, is often linked to improved colloidal stability because of strong electrostatic repulsion between particles, it can also lead to unintended effects on biological interactions, like potentially lowering biodistribution and targeting efficiency.^66,67^

Nevertheless, achieving even narrower size distributions remains a significant challenge for nanoparticle tracers. Previous studies have shown that optimization strategies; such as extended or probe ultrasonication, careful adjustment of pH and ionic strength, addition of steric stabilizers, and postsynthesis size selection through filtration or differential centrifugation; can effectively reduce aggregation and improve the monodispersity and stability of iron-oxide nanoparticles.^68–70^ Applying these strategies in future work could enhance the physicochemical properties of ZnFe_2_O_4_ nanoparticles, increasing their potential for biomedical imaging applications.

### 3.5 TGA Results

The impact of different surfactant types during the hydrothermal process for coating agents on particle surfaces was studied using a Thermogravimetric Analyzer (TGA). Figure 7 displays the TGA curves of coated ZnFe_2_O_4_ samples. Three weight loss steps were observed during heat treatment. The initial degradation curve of the samples showed a weight loss percentage of approximately 1%, 1.95%, and 1.58% at temperatures of 104 °C, 128 °C, and 120 °C for CA, AA, and TA coated samples, respectively. This first loss can be attributed to water adsorbed on the surface of the nanoparticles. Following the removal of the remaining water molecules, a more significant degradation occurred, with a weight loss percentage of about 24.6% (104–319 °C), 2.3% (128–453 °C), and 4.2% (120–463 °C) for the CA, AA, and TA-coated ZnFe_2_O_4_ samples, respectively. More than half of the weight loss occurred during this stage, where the majority of organic matter decomposes, primarily due to the loss of the carboxylate (-COOH) group.^71^

**Figure 7:**
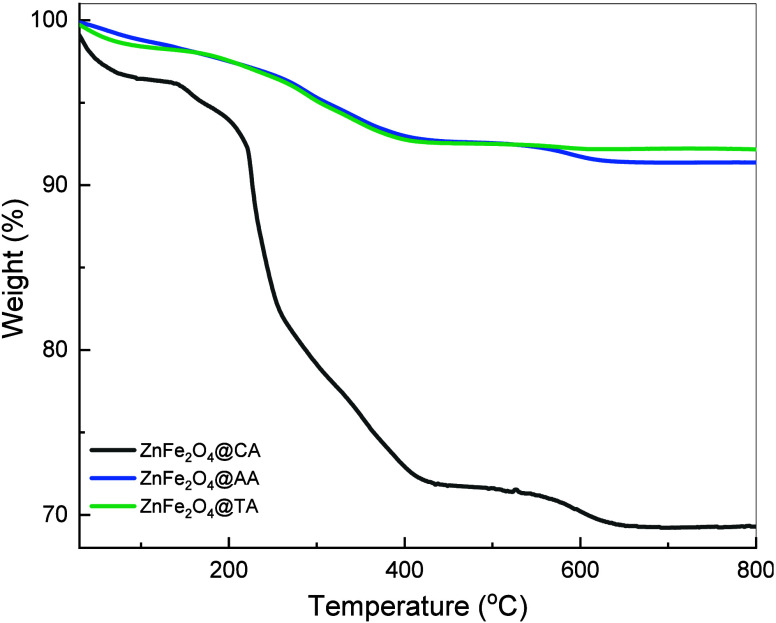
TGA patterns of ZnFe_2_O_4_@CA, ZnFe_2_O_4_@AA, and ZnFe_2_O_4_@TA nanoparticles.

The minor final weight loss observed at higher temperatures can be attributed to the removal of oxygen functionalities and residual functional groups. CA, AA, and TA-coated samples show a loss percentage of approximately 2.1%, 3.2%, and 1.3% (at temperatures from 319 to 458 °C, 453 to 697 °C, and 463 to 668 °C), respectively. The TA-coated sample exhibits minimal alteration (approximately 7% weight loss) across a temperature range of 100–800 °C, indicating significant temperature and phase stability. However, the CA-coated ZnFe_2_O_4_ sample is observed to be more thermally unstable (about 28% weight loss) than the other samples. This a notable mass loss suggests either a relatively thick organic layer exists or that not all precursor residues have been completely removed. Furthermore, the extensive, sequential degradation steps seen in ZnFe_2_O_4_@CA imply that secondary phases could also develop during the synthesis process, decomposing at different temperature ranges. This occurrence may stem from the strong chelating ability of citric acid, which could help with complexation and phase segregation, reinforcing our findings from XRD and FTIR.

## 4 Magnetic Analysis Results and Discussion

### 4.1 ESR Results

To learn more about the spin dynamics of nanosized systems, the EPR spectra were recorded at a frequency of 9.11 GHz at room temperature (300 K). Figure 8 shows typical ESR patterns for uncoated and CA, AA, and TA-coated ZnFe_2_O_4_ nanoparticles, which consist of a single, broad line in the relevant temperature range. It is noted that all samples contain unpaired electrons and display magnetic behavior. An examination of Figure 8 indicates that coated samples have similar intensity values, with significant changes observed in the intensity peaks compared to the uncoated sample. The intensity value of the uncoated ZnFe_2_O_4_ nanoparticle is 7053 au. The decreased intensity peak values of nanoparticles with coated surfactants indicate an effect on the magnetic properties of the samples.

**Figure 8:**
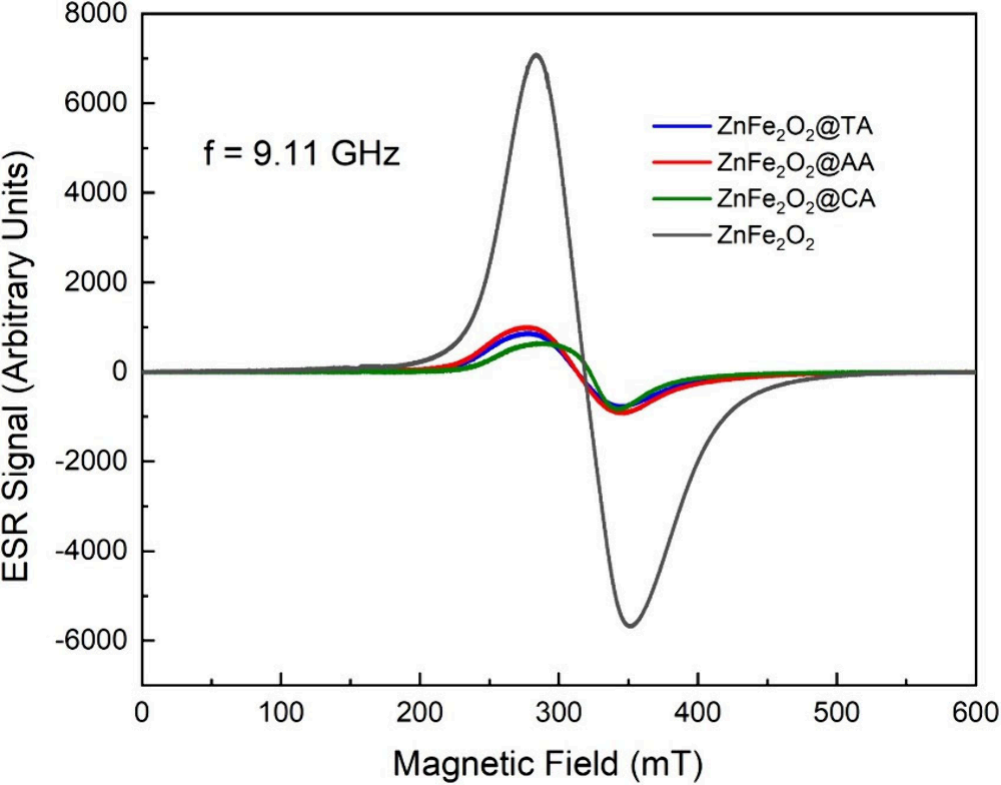
ESR spectra of bare ZnFe_2_O_4_, ZnFe_2_O_4_@CA, ZnFe_2_O_4_@TA, and ZnFe_2_O_4_@AA nanoparticles.

The main parameters, including *T*_*2*_: relaxation times of the synthesized samples, *N*_*s*_: the number of unpaired electrons’ spins, *I*_pp_: the signal intensities from peak to peak, the Δ*H*_pp_: peak-to-peak line widths of the ESR-active spins, Δ*H*_1/2_: Full-width half-maximum, *g*: Lande factor, and *P*_asy_: the asymmetry factor were calculated using ESR equations.^72,73^ The analytical results are depicted in Table 3. Δ*H*_1/2_ was calculated using the formula in eq 7:

7 $$\Delta H_{1/2}=\sqrt{3}\times \Delta H_{pp}$$

**Table 3:** ESR Parameters of Uncoated ZnFe_2_O_4_, ZnFe_2_O_4_@CA, ZnFe_2_O_4_@AA and ZnFe_2_O_4_@TA nanoparticles

sample no.	*g*-value	*Δ* *H*_p__p_ (mT)	*I*_p__p_ (10^4^)	*N*_s_ (10^8^)	*T*_2_ (10^–11^ s)	*P*_a__*s*__y_
ZnFe_2_O_4_	2.04	70.04	582	814	4.58	2.2
ZnFe_2_O_4_@CA	2.02	58.27	500	484	5.57	1.7
ZnFe_2_O_4_@AA	2.07	65.39	121	148	4.83	2.0
ZnFe_2_O_4_@TA	2.07	65.39	165	201	4.83	2.1

The intensity of the ESR peaks varies with the coating agents, but the peak location and broadness of the signals remain constant. The uncoated ZnFe_2_O_4_ sample shows the strongest signal. The line width Δ*H*_pp_ values were obtained by the distance between the derivative of the ESR peak’s maximum and minimum values. The AA-coated sample exhibits the highest line width among the coated samples.

The quantity of spins provides insights into the unpaired electrons that contribute to the resonance signal. The total number of unpaired electrons in each of the generated samples was calculated using eq 8:

8 $$N_{s}=0.285\times I_{pp}\times \Delta {H^{2}}_{pp}$$

The highest value was observed for uncoated ZnFe_2_O_4_ nanoparticles. The number of unpaired electrons reduced as coatings were added. The *g* value determined from the magnetic field and frequency measurements when the resonance occurs, using eq 9:

9 $$g=\frac{hf}{\beta B}$$

where *h* is Planck constant, *f* is the frequency (9.11 GHz) of microwave, β is the Bohr magneton, and *B* is the resonance magnetic field. The *g*-value obtained for all synthesized samples is very close to that of a free electron (∼2.0023). These results suggest that the magnetic properties of the synthesized materials resemble those of a free electron.^72^ Among the different coated surfactants, the lower *g*-value is observed only for the CA-coated sample compared to the bare zinc ferrite.

In the differential ESR line shape, *P*_asy_ is defined as the ratio of the positive maximum to the negative maximum.^73,74^ This parameter provides insights into the homogeneity of the sample. The asymmetry parameters of the prepared samples were determined using the following eq 10:

10 $$P_{asy}=1-\frac{h_{u}}{h_{L}}$$

The absorption peak heights of ESR spectra above and below the baseline are represented by the letters *h*_u_ and *h*_L_. Findings show that adding coating agents decreases asymmetry parameters, and the minimum value was observed for the CA-coated sample. eq 11 was used to determine the spin–spin relaxation time constant, *T*_*2*__:_

11 $$\frac{1}{T_{2}}=\frac{g\beta \Delta H_{1/2}}{\hbar }$$

As it is noted that the spin–spin relaxation time (*T*_2_) of excited electrons is inversely related to the line width Δ*H*_1/2_. The observed increase in relaxation time on the surface-modified samples can be linked to the weakened spin flips (exchange interaction).^74^

### 4.2 PPMS Result

The PPMS technique evaluated the magnetic properties of the synthesized uncoated and CA, AA, and TA-coated ZnFe_2_O_4_ samples. Figure 9 shows the magnetization curves of all samples from approximately −60 kOe to +60 kOe, recorded at 10K, 300 K, and 400 K, respectively.

**Figure 9:**
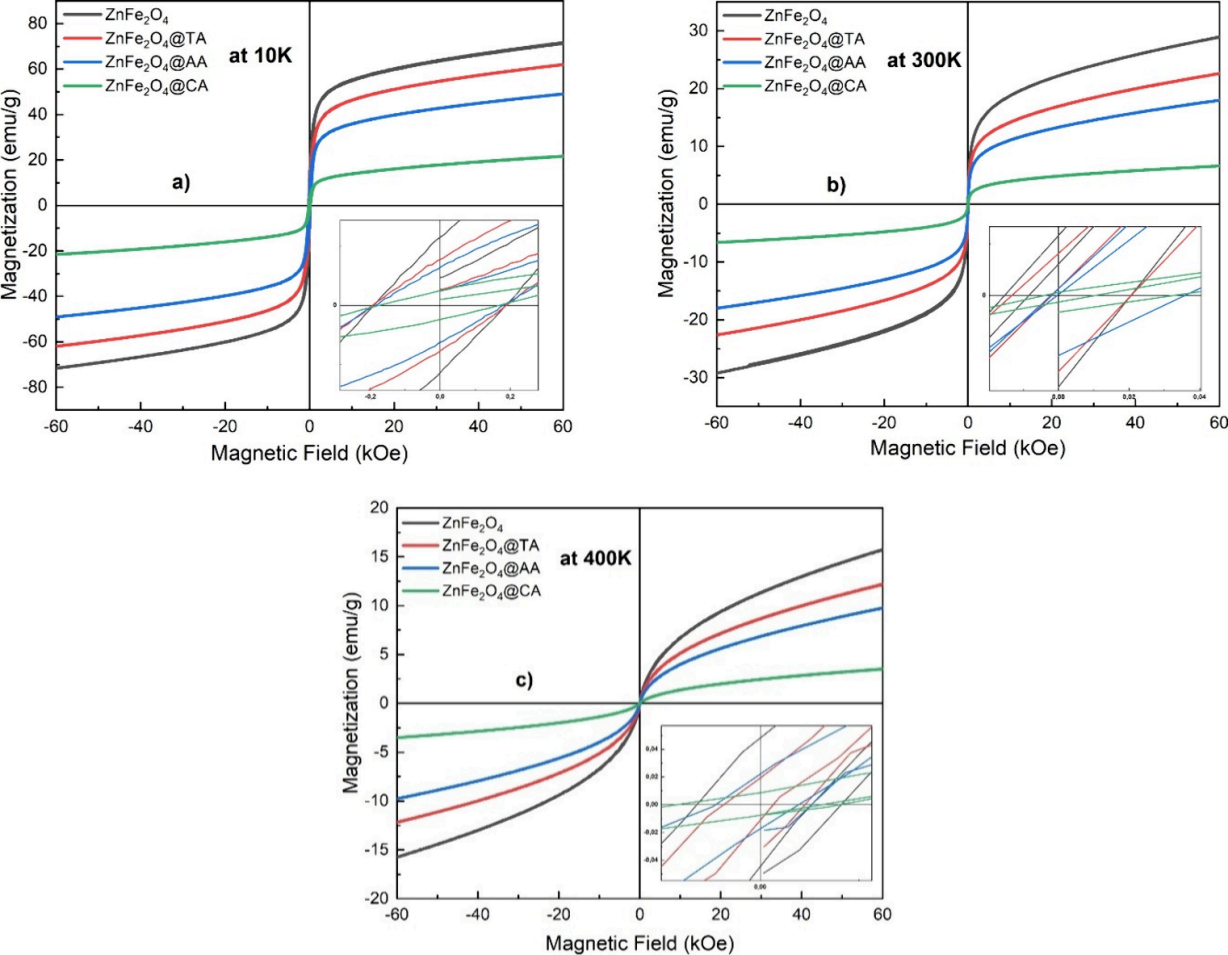
Magnetization curves of uncoated ZnFe_2_O_4_, ZnFe_2_O_4_@TA, ZnFe_2_O_4_@AA, and ZnFe_2_O_4_@CA nanoparticles at (a) 10 K, (b) 300 K, and (c) 400 K are shown, with the insets providing a magnified view of the hysteresis loops near zero magnetic field, highlighting the coercivity and remanence.

As seen in the focused insets of Figure 9, the samples exhibited a negligible hysteresis, i.e., insignificant remanence and minimal coercivities at all measured temperatures, indicating superparamagnetic and single-domain nanoparticles. The hysteresis loop reflects the magnetic characteristics of the samples, including saturation magnetization (*M*_s_), remanent magnetization (*M*_r_), and coercivity (*H*_c_), which are shown in Table 4. The value of saturation magnetization *M*_s_ depends on grain size, coating agents, and preparation methods, particularly its temperature value.^30,75,76^ The saturation magnetization values are approximately 21–71, 6–28, and 3–15 emu/g at 10 K, 300 K, and 400 K, respectively.^51^ The results indicate that the saturation magnetization (*M*_s_) values of the nanoparticles decreased with the incorporation of coating agents across all varying reaction temperatures. The lowest *M*_s_ value is observed for CA-coated zinc ferrite samples. There may be several reasons for this phenomenon. First, the decrease in *M*_s_ values of the coated samples can be attributed to a nonmagnetic layer on the surfaces of the samples. Surface structural distortions in coated ZnFe_2_O_4_ nanoparticles may result in the reduction of *M*_s_ values. Moreover, *M*_s_ of coated ZnFe_2_O_4_ nanoparticles gradually decreases with the change in particle size due to spin configuration or spin canting effects.^77^ For PPMS analysis, the samples were collected using a magnet and dried at 60 °C for 2 h in an oven before characterization, until the sample is considered dry. While we cannot entirely exclude a small diamagnetic contribution a weak linear background observed, especially in Figure 9b and c (300 and 400 K).

**Table 4:** PPMS Parameters at 10 K, 300 K, and 400 K for the Synthesized Material Samples

Measurement Temp	Sample	*M*_s_ (emu/g)	*M*_r_ (emu/g)	*H*_c_ (Oe)	SQR (*M*_r_/*M*_s_)	*K* (J/m^3^)	*n*_B_ (μ_B_)
10 K	ZnFe_2_O_4_	71	0.18	3	0.002	332	3.06
	ZnFe_2_O_4_@TA	62	0.08	3	0.001	290	2.67
	ZnFe_2_O_4_@AA	49	0.14	5	0.002	382	2.11
	ZnFe_2_O_4_@CA	21	0.08	2	0.003	65	0.9
300 K	ZnFe_2_O_4_	28	0.3	5	0.010	218	1.20
	ZnFe_2_O_4_@TA	22	0.33	7	0.015	240	0.94
	ZnFe_2_O_4_@AA	18	0.03	33	0.001	928	0.77
	ZnFe_2_O_4_@CA	6	0.03	6	0.005	56	0.25
400 K	ZnFe_2_O_4_	15	0.004	3	0.0002	70	0.64
	ZnFe_2_O_4_@TA	12	0.007	8	0.0005	150	0.51
	ZnFe_2_O_4_@AA	9	0.01	1	0.001	14	0.38
	ZnFe_2_O_4_@CA	3	0.001	5	0.0003	23	0.13

The *M*_r_ values of the samples are negligible in the range of 0.08–0.18, 0.03–0.33, and 0.007–0.01 emu/g at 10 K, 300 K, and 400 K, respectively. Similarly, the coercivity (*H*_c_) of the synthesized samples was also found to be negligible, with values ranging from 2 to 5 Oe at 10 K, 5–33 Oe at 300 K, and 1–8 Oe at 400 K, further supporting the superparamagnetic behavior. The minor change in coercivity and remanent magnetization of CA, AA, and TA-coated ZnFe_2_O_4_ nanoparticles may be associated with the change in size and crystallinity of the samples.^78^

The average magnetic diameter (*D*_M_) were calculated by fitting the Langevin equation and found between 13.5 and 20 nm. Those *D*_M_ values are following the values calculated using XRD data. The squareness ratio ( SQR=MrMs ) provides information regarding whether nanoparticles are multiple magnetic domains or single domains. The calculated SQR values of all samples were determined between 0.001 and 0.015, listed in Table 4. These values demonstrate that the nanoparticles relax so fast, like a superparamagnet at RT, even without an external magnetic field.^79^

The magnetic moment per formula unit (*n*_*B*_) for the compositions has been calculated by using the following formula: $n_{B}=\frac{M_{w}xM_{s}}{5585}$ , where *M*_*w*_ is the molecular weight. The calculated *n*_B_ values for all samples are in the range of 0.9–3.06, 0.25–1.20, and 0.13–0.64 at 10 K, 300 K, and 400 K, respectively. As it is seen in the formula, *n*_B_ is linearly proportional to the value of saturation magnetization. Hence, the increased *n*_B_ values are observed with the higher *M*_s_ valued samples.

Magnetic anisotropy refers to the directional dependence of a material’s magnetic properties, with the anisotropy constant (*K*) quantifying the strength of this dependence within a given crystal structure. The anisotropy constants are calculated using the following equation: $K=\frac{\mu_{o}\times MsxHc}{2}$ , where μ_o_ is the Bohr magneton. *K* values decrease with the inclusion of a surface coating layer and follow the trends of *H*_c_ values since the anisotropy strongly contributes to the *H*_c_ *. K* values were found in the range of 65–382, 56–928, and 14–150 J/m^3^ at 10, 300, and 400 K, respectively. All those PPMS results listed in Table 4 aligned with ESR findings.

The temperature-dependent behavior of the zero-field cooling (ZFC) and field cooling (FC) curves for the CA, AA, and TA-coated ZnFe_2_O_4_ nanoparticles were examined under an applied magnetic field to provide deeper insights into the magnetic behaviors of the samples. For ZFC magnetization, the sample is initially cooled to 10K without applying a magnetic field. Subsequently, a magnetic field is introduced, and the magnetization is recorded while the sample is heated to 400 K. In contrast, FC magnetization is measured by cooling the sample from 400 K to 10K in the presence of the applied field. Figure 10 presents the ZFC and FC magnetization curves obtained over a temperature range from 10K to 400 K. As illustrated in the Figure 10, ZFC and FC curves exhibit overlap at elevated temperatures, subsequently diverging substantially as the temperature decreases.

**Figure 10:**
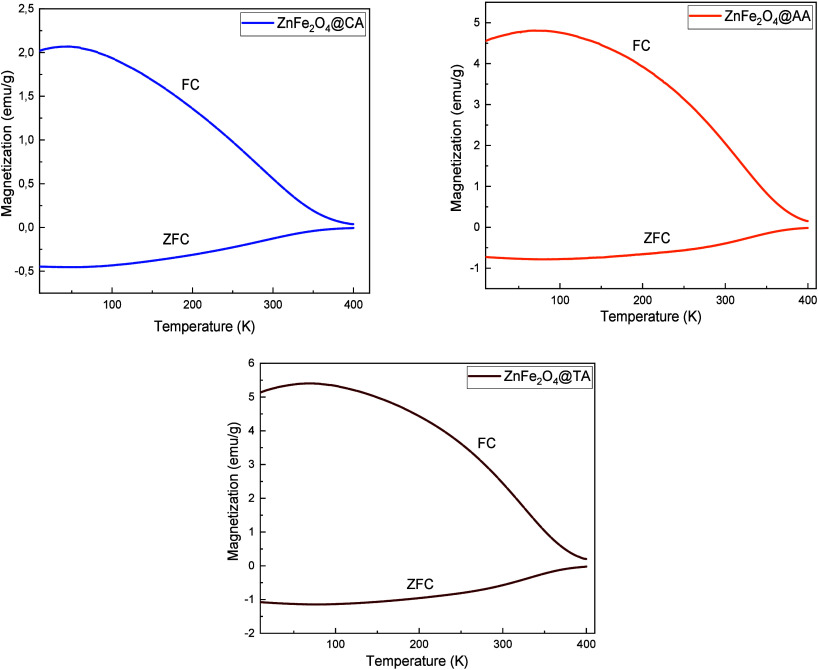
Magnetization versus temperature plots of ZnFe_2_O_4_@CA, ZnFe_2_O_4_@AA, and ZnFe_2_O_4_@TA nanoparticles.

In a ZFC curve, magnetization gradually increases with rising temperature until it reaches the point of vanishing magnetization, which corresponds to the blocking temperature (*T*_B_). At this temperature, thermal energy (*k*_B_ *T*) becomes comparable to the magnetic anisotropy energy (*K*_eff_ *V*). The blocking temperature plays a critical role in the characterization of particles exhibiting uniaxial anisotropy, and is defined as the temperature at which the average time for a magnetic nanoparticle’s moment to escape from the energy well equals the measurement time of the system.^80^ Below this temperature, the particles remain thermally stable. As shown in Figure 10, the ZFC and FC curves start to coincide at a blocking temperature of approximately 400 K, indicating that all nanoparticles are in the same superparamagnetic state. In addition, the reduction in magnetization at elevated temperatures may be attributed to the superparamagnetic response of distinct magnetic clusters, which can exhibit ferromagnetic behavior.^51^

### 4.3 MPS Results

This study presents the synthesis and characterization of CA, AA, and TA-coated ZnFe_2_O_4_ nanoparticles, emphasizing their potential as tracer agents in magnetic particle imaging (MPI) systems. MPS is based on detecting the nonlinear magnetic susceptibility of magnetic nanoparticles and can provide dynamic magnetic properties of samples, which is significant in determining the performance of tracers in MPI.^81^ The detected spectrum comprises odd harmonics of the excitation frequency generated by the nanoparticles. The amplitudes of the lower harmonics are proportional to both the number of particles and their signal yield, while higher harmonics indicate the spatial resolution that can be attained. In this study, MPS characterization of the uncoated, CA, AA, and TA-coated ZnFe_2_O_4_ nanoparticles were performed with a homemade relaxometer at 9.9 kHz with a 15 mT sinusoidal excitation field.^39,40^ The key properties were assessed, including relaxation time, resolution (FWHM in mT), and relative signal strength. The results were subsequently compared with the most frequently utilized reference tracer materials for magnetic particle imaging (MPI), which include Perimag (Micromod GmbH, Germany) and Vivotrax (Magnetic Insight, USA).

The recorded odd harmonics FFT spectrum of ZnFe_2_O_4_ samples and reference tracers is presented on Figure 11. The slow decay rate of higher harmonics in the spectra serves as a criterion for identifying optimal nanoparticles. Compared to reference materials, all synthesized samples demonstrate a faster response to the excitation field at lower relaxation times, as shown in Table 5. Here, we used the harmonic ratio 5th/3rd (representing the spectrum’s shape) to assess changes in the MPS signal. CA-coated ZnFe_2_O_4_ exhibits a fast-decaying FFT response, and the shortest relaxation time compared to other coated ZnFe_2_O_4_ samples.

**Figure 11:**
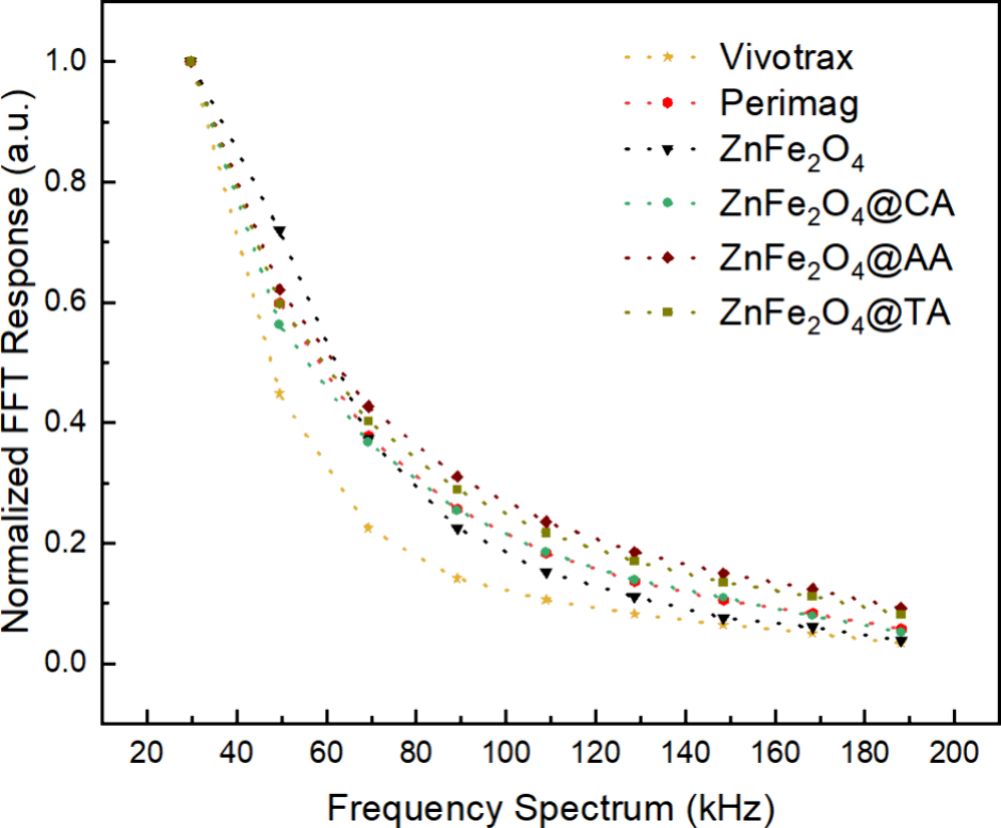
Normalized odd harmonic levels for reference (Perimag and Vivotrax) and synthesized nanoparticles.

**Table 5:** Comprehensive Evaluation of ZnFe_2_O_4_ Nanoparticles Based on MPS Measurements

Sample	5th/3rd	FWHM (mT)	relaxation time (μs)
Perimag	0.62	5.70	4.5 ^43^
Vivotrax	0.44	9.05	4.44 ^43^
ZnFe_2_O_4_	0.719	6.37	3.67 ^43^
ZnFe_2_O_4_@CA	0.563	5.89	2.09
ZnFe_2_O_4_@AA	0.621	5.14	2.85
ZnFe_2_O_4_@TA	0.597	5.28	3.18

The effective relaxation time characterizes the response time of nanoparticles to an alternating magnetic field at a specific frequency, which is a critical parameter in determining the size limit of potential tracers for MPI imaging. A lower relaxation time is highly desirable for MPI tracer agents, enabling a more rapid magnetic response. The effective relaxation times of the synthesized ZnFe_2_O_4_ nanoparticles were evaluated and compared with those of standard reference materials. Results, summarized in Table 5, indicate that all synthesized ZnFe_2_O_4_ nanoparticles exhibit shorter relaxation times than the reference tracers (Vivotrax, Perimag), reflecting a faster response to the applied magnetic field. This result highlights the potential of the synthesized nanoparticles for quantitative MPI applications.

The point spread function (PSF) serves as a key performance metric, describing how magnetization responds to the applied excitation field. In the X-space (time-domain) reconstruction technique, the PSF of the samples is utilized instead of the FFT response. The resolution in MPS relaxometer systems is conventionally defined by the full width at half-maximum (FWHM), where a narrower PSF width corresponds to higher resolution. The MPS performance of the synthesized samples is characterized by the tracer response fwhm of the normalized PSFs, as illustrated in Figure 12. Samples with the highest normalized signal intensity and the narrower FWHM exhibit the best spatial resolution. As shown in Figure 12, all synthesized ZnFe_2_O_4_ nanoparticles demonstrate better resolution than Perimag and Vivotrax. The optimized uncoated and coated ZnFe_2_O_4_ samples have much narrower and distinct peaks, indicative of significantly better MPS performances compared to Vivotrax and Perimag, which serve as reference materials. The longer relaxation time observed in the uncoated ZnFe_2_O_4_ sample, despite its smaller hydrodynamic size, is likely due to aggregation and poor colloidal stability. All magnetic nanoparticles consist of a magnetic core and a hydrodynamic (nonmagnetic) coating. Without such a stabilizing coating, uncoated nanoparticles tend to cluster and agglomerate due to strong interparticle magnetic and van der Waals interactions. This aggregation restricts Brownian motion and introduces interparticle magnetic coupling, both of which effectively slow down the overall magnetic relaxation process. Furthermore, the formation of these clusters leads to a distribution of sizes and magnetic behaviors that impair the predictability and consistency of their physicochemical and magnetic dynamics. In contrast, surface coatings—such as citric acid (CA), ascorbic acid (AA), or tartaric acid (TA)—provide colloidal stability, prevent aggregation, and maintain a uniform hydrodynamic size. This allows coated ZnFe_2_O_4_ nanoparticles to remain dispersed in suspension, supporting faster and more independent relaxation through both Néel and Brownian mechanisms. In our study, although all coated ZnFe_2_O_4_ samples outperformed commercial tracers like Perimag and Vivotrax in both fwhm and relaxation time, ZnFe_2_O_4_@AA showed the best overall performance, with the lowest FWHM (5.14 mT) and the highest 5th/3rd harmonic ratio (0.621), indicating a stronger signal and better resolution. For applications that prioritize maximum spatial resolution and signal strength, such as cell tracking or lesion detection, ZnFe_2_O_4_@AA is recommended. If broader dispersion stability or a slightly faster response across other metrics is needed, CA- or TA-coated versions may still be suitable. Recent works support this guidance: flame-made Zn-doped iron oxide tracers with citrate coatings have shown exceptional FWHM and relaxation time performance among sub-20 nm particles, confirming that both coating composition and hydrodynamic size distribution are critical for MPI tracer performance.^82–84^

**Figure 12:**
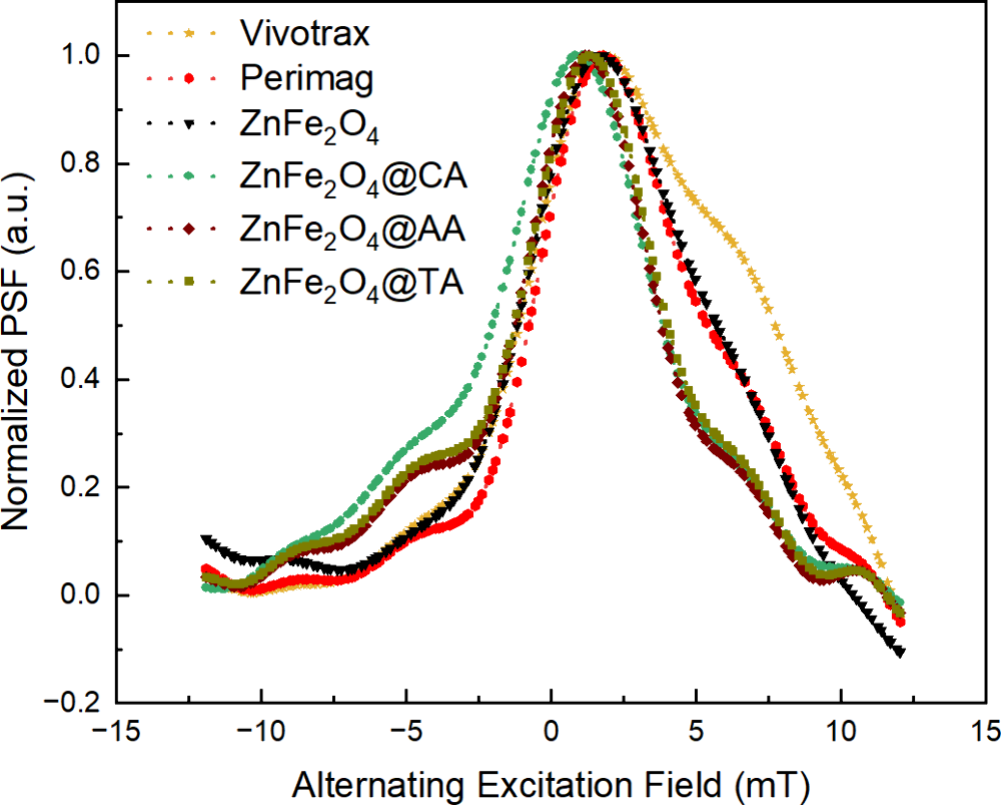
Normalized PSFs of the Vivotrax, Perimag, bare, and coated ZnFe_2_O_4_ nanoparticles.

## 5 Conclusion

MPI represents a groundbreaking imaging technique that facilitates tomographic imaging and quantifies superparamagnetic tracers spread throughout a 3D space. In this study, we explored the effect of adding coating agents to naked ZnFe_2_O_4_ nanoparticles and evaluated their performance as tracer agents for MPI systems. ZnFe_2_O_4_-based magnetic nanoparticles, coated with biocompatible agents CA, AA, and TA, were successfully prepared using hydrothermal and coprecipitation methods and were characterized using structural and magnetic evaluation techniques. The FTIR spectra established the presence of coating agents and their effects on the ZnFe_2_O_4_ nanoparticles, referring to the chemical chains. XRD analysis confirmed that ZnFe_2_O_4_ has a face-centered cubic (FCC) structure with an average core particle size of 14 nm, while the use of coatings led to an increase in particle size. It was found that coating agents significantly affected the nanoparticles’ dislocation and specific surface area parameters. SEM analysis showed that the nanoparticles are near-spherical, and EDX analysis confirmed that they contain no other impurities. DLS analysis confirmed that the samples are in a colloidally stable form. ESR and PPMS results confirm that the samples’ behavior is superparamagnetic. The coating agents reduced the magnitude of saturation magnetization and increased the magnetic coercivity. The particle size influenced magnetic behaviors, with the coercivity value rising as the particle size increases. MPS measurements showed that all coated ZnFe_2_O_4_ nanoparticles outperformed commercial tracers (Perimag and Vivotrax) in relaxation time and spatial resolution, with AA-coated ZnFe_2_O_4_ demonstrating the best overall MPS performance (FWHM 5.14 mT, relaxation time 2.85 μs). These results emphasize the significance of coating chemistry in balancing stability, magnetic responsiveness, and surface interactions. We clarify that our current zeta potential measurements were performed in deionized water, which confirms intrinsic surface charge stabilization but does not replicate physiological ionic strength. In physiological media such as PBS or serum, high ionic strength compresses the electrical double layer, which can reduce electrostatic repulsion and promote aggregation. Besides, it is essential to acknowledge that a rigorous comparison would require assessing CA, TA, and AA-coated nanoparticles under identical size distributions. This will demonstrate that dependency of MPS tracer efficiency on particle size and the interfacial chemistry of the coating, how can significantly affect relaxation behavior and spatial resolution. Future studies will therefore focus on testing colloidal stability in different biological media, while also investigating molecular-level interactions at the nanoparticle–coating interface and their impact on magnetic dynamics, with the overarching goal of refining coating strategies that optimize both magnetic performance and biological compatibility for next-generation MPI tracers.

## References

[1] Gleich B., Weizenecker J. (2005). Tomographic imaging using the nonlinear response of
magnetic particles. Nature.

[2] Pablico-Lansigan M. H., Situ S. F., Samia A. C. S. (2013). Magnetic
particle imaging: advancements
and perspectives for real-time in vivo monitoring and image-guided
therapy. Nanoscale..

[3] Kannan, M. Krishnan. Fundamentals and Applications of Magnetic Materials; Oxford University Press, 2016.

[4] Vogel P., Ruckert M. A., Friedrich B., Tietze R., Lyer S., Kampf T., Hennig T., Dolken L., Alexiou C., Behr V. C. (2022). Critical Offset
Magnetic particle spectroscopy for
rapid and highly sensitive medical point-of-care diagnostics. Nat. Commun..

[5] Malhotra A., von Gladiss A., Behrends A., Friedrich T., Neumann A., Buzug T. M., Lüdtke-Buzug K. (2019). Tracking the
growth of superparamagnetic nanoparticles with an in-situ magnetic
particle spectrometer (INSPECT). Sci. Rep..

[6] Elfers P. N., Ludtke-Buzug K., Malhotra A., Ackers J., Mirzojan L., Wattenberg M., Engster J. C., Melenberg D., Ahlborg M., Friedrich T., Buhne M.-J., Sieren M. M., Buzug T. M., Kloeckner R., Barkhausen J., Wegner F. (2025). Development and Degradation Study
of PLA-Based Medical
Implant Markers for Magnetic Particle Imaging. Macromol. Biosci..

[7] Knopp, T. ; Buzug, T. M. Magnetic Particle Imaging An Introduction to Imaging Principles and Scanner Instrumentation. SpringerLink: Berlin; 2012.

[8] Goodwill P.
W., Saritas E. U., Croft L. R., Kim T. N., Krishnan K. M., Schaffer D. V. (2012). X-Space MPI: Magnetic Nanoparticles for Safe
Medical Imaging. Adv. Mater..

[9] Weizenecker J., Borgert J., Gleich B. (2007). A simulation
study on the resolution
and sensitivity of magnetic particle imaging. Phys. Med. Biol..

[10] Hufschmid R., Landers J., Shasha C., Salamon S., Wende H., Krishnan K. M. (2019). Nanoscale Physical
and Chemical Structure of Iron Oxide
Nanoparticles for Magnetic Particle Imaging. Physica Status Solidi A.

[11] Wu K., Su D., Saha R., Liu J., Chugh V. K., Wang J. P. (2020). Magnetic
Particle Spectroscopy: A Short Review of Applications Using Magnetic
Nanoparticles. ACS Appl. Nano Mater..

[12] Israel L. L., Galstyan A., Holler E., Ljubimova J. Y. (2020). Magnetic
iron oxide nanoparticles for imaging, targeting and treatment of primary
and metastatic tumors of the brain. J. Controlled
Release.

[13] Kaman O., Kubániová D., Kubíčková L., Herynek V., Veverka P., Jirák Z. (2024). Magnetic particle spectroscopy and magnetic particle imaging of zinc
and cobalt ferrite nanoparticles: Distinct relaxation mechanisms. J. Alloys Compd..

[14] Pucci C., Degl’Innocenti A., Belenli Gümüs M., Ciofani G. (2022). Superparamagnetic iron oxide nanoparticles for magnetic
hyperthermia: recent advancements, molecular effects, and future directions
in the omics era. Biomater Sci..

[15] Ansari M.
O., Ahmad M. F., Shadab G.G.H.A., Siddique H. R. (2018). Superparamagnetic
iron oxide nanoparticles based cancer theranostics: A double edge
sword to fight against cancer. J. Drug Deliv
Sci. Technol..

[16] Samrot A. V., Sahithya C. S., Selvarani A. J., Purayil S. K., Ponnaiah P. (2021). A review on
synthesis, characterization and potential biological applications
of superparamagnetic iron oxide nanoparticles. Current Research in Green and Sustainable Chemistry..

[17] Laurent, S. ; Boutry, S. ; Muller, R. N. Metal Oxide Particles and Their Prospects for Applications. In Iron Oxide Nanoparticles for Biomedical Applications; Elsevier; 2018; pp 3–42.

[18] Li Y., Wang N., Huang X., Li F., Davis T. P., Qiao R. (2020). Polymer-Assisted Magnetic
Nanoparticle Assemblies for
Biomedical Applications. ACS Appl. Bio Mater..

[19] Raeisi
Shahraki R., Ebrahimi M., Seyyed Ebrahimi S. A., Masoudpanah S. M. (2012). Structural characterization and magnetic properties
of superparamagnetic zinc ferrite nanoparticles synthesized by the
coprecipitation method. J. Magn Magn Mater..

[20] Sahoo P., Choudhary P., Laha S. S., Dixit A., Mefford O. T. (2023). Recent
advances in zinc ferrite (ZnFe_2_O_4_) based nanostructures
for magnetic hyperthermia applications. Chemical
Communications..

[21] Atif M., Hasanain S. K., Nadeem M. (2006). Magnetization
of sol–gel prepared
zinc ferrite nanoparticles: Effects of inversion and particle size. Solid State Commun..

[22] Hasirci C., Karaagac O., Köçkar H. (2019). Superparamagnetic
zinc
ferrite: A correlation between high magnetizations and nanoparticle
sizes as a function of reaction time via hydrothermal process. J. Magn Magn Mater..

[23] Manikandan A., Kennedy L. J., Bououdina M., Vijaya J. J. (2014). Synthesis, optical
and magnetic properties of pure and Co-doped ZnFe_2_O_4_ nanoparticles by microwave combustion method. J. Magn Magn Mater..

[24] Ebrahimi M., Raeisi Shahraki R., Seyyed Ebrahimi S. A., Masoudpanah S. M. (2014). Magnetic
Properties of Zinc Ferrite Nanoparticles Synthesized by Coprecipitation
Method. J. Supercond Nov Magn..

[25] Tomar D., Jeevanandam P. (2022). Synthesis of ZnFe2O4 nanoparticles with different morphologies
via thermal decomposition approach and studies on their magnetic properties. J. Magn Magn Mater..

[26] Pemartin K., Solans C., Alvarez-Quintana J., Sanchez-Dominguez M. (2014). Synthesis
of Mn–Zn ferrite nanoparticles by the oil-in-water microemulsion
reaction method. Colloids Surf. A Physicochem
Eng. Asp..

[27] Cobos M. A., de la Presa P., Llorente I., García-Escorial A., Hernando A., Jiménez J. A. (2020). Effect of preparation methods on
magnetic properties of stoichiometric zinc ferrite. J. Alloys Compd..

[28] Sonia L. C., Victory M., Phanjoubam S. (2020). A Comparative Study of the Properties
of Zinc Ferrite Nanoparticles Synthesized by Different Techniques
for Nanofluid Preparation. Integr. Ferroelectr..

[29] Zargar T., Kermanpur A. (2017). Effects of hydrothermal process parameters on the physical,
magnetic and thermal properties of Zn_0.3_Fe_2.7_O_4_ nanoparticles for magnetic hyperthermia applications. Ceram. Int..

[30] Carvalho M. D., Henriques F., Ferreira L. P., Godinho M., Cruz M. M. (2013). Iron oxide
nanoparticles: the influence of synthesis method and size on composition
and magnetic properties. J. Solid State Chem..

[31] da Silva S, P. ; de Moraes D, C. Iron Oxide Nanoparticles Coated with Polymer Derived from Epoxidized Oleic Acid and Cis-1,2-Cyclohexanedicarboxylic Anhydride: Synthesis and Characterization. Journal of Material Science & Engineering 2016, 5(3). 10.4172/2169-0022.1000247.

[32] Liu S., Yu B., Wang S., Shen Y., Cong H. (2020). Preparation, surface
functionalization and application of Fe_3_O_4_ magnetic
nanoparticles. Adv. Colloid Interface Sci..

[33] Behdadfar B., Kermanpur A., Sadeghi-Aliabadi H., del Puerto Morales M., Mozaffari M. (2012). Synthesis
of aqueous ferrofluids of ZnxFe3–xO4
nanoparticles by citric acid assisted hydrothermal-reduction route
for magnetic hyperthermia applications. J. Magn
Magn Mater..

[34] Xuan S., Hao L., Jiang W., Gong X., Hu Y., Chen Z. (2007). Preparation
of water-soluble magnetite nanocrystals through hydrothermal approach. J. Magn Magn Mater..

[35] Yan J., Mo S., Nie J., Chen W., Shen X., Hu J. (2009). Hydrothermal
synthesis of monodisperse Fe3O4 nanoparticles based
on modulation of tartaric acid. Colloids Surf.
A Physicochem Eng. Asp..

[36] Nangare S., Vispute Y., Tade R., Dugam S., Patil P. (2021). Pharmaceutical
applications of citric acid. Future Journal
of Pharmaceutical Sciences..

[37] Hastak V., Bandi S., Kashyap S., Singh S., Luqman S., Lodhe M., Peshwe D. R., Srivastav A. K. (2018). Antioxidant
efficacy of chitosan/graphene functionalized superparamagnetic iron
oxide nanoparticles. J. Mater. Sci.: Mater.
Med..

[38] Scharlach C., Kratz H., Wiekhorst F., Warmuth C., Schnorr J., Genter G., Ebert M., Mueller S., Schellenberger E. (2015). Synthesis
of acid-stabilized iron oxide nanoparticles and comparison for targeting
atherosclerotic plaques: evaluation by MRI, quantitative, MPS, and
TEM alternative to ambiguous Prussian blue iron staining. Nanomedicine: Nanotechnology, Biology and Medicine..

[39] Irfan M., Dogan N., Sapmaz T., Bingolbali A. (2021). Development
of MPI relaxometer for characterization of superparamagnetic nanoparticles. J. Magn Magn Mater..

[40] Irfan M., Dogan N. (2022). Comprehensive Evaluation of Magnetic Particle Imaging (MPI) Scanners
for Biomedical Applications. IEEE Access..

[41] Paysen H., Wells J., Kosch O., Steinhoff U., Trahms L., Schaeffter T., Wiekhorst F. (2018). Towards quantitative
magnetic particle imaging: A comparison with magnetic particle spectroscopy. AIP Advances.

[42] Yin L., Li W., Du Y., Wang K., Liu Z., Hui H., Tian J. (2022). Recent developments of the reconstruction in magnetic particle imaging. Visual computing for industry, biomedicine, and art.

[43] Dogan N., Caliskan G., Irfan M. (2023). Synthesis
and characterization of
biocompatible ZnFe_2_O_4_ nanoparticles for magnetic
particle imaging (MPI). Journal of Materials
Science: Materials in Electronics..

[44] Ozer S., Dogan N., Canim-Ates S., Bingolbali A. (2025). Synthesis
and Characterization of Coated CoFe_2_O_4_ Nanoparticles
with Biocompatible Compounds and In Vitro Toxicity Assessment on Glioma
Cell Lines. Advanced Materials Interfaces..

[45] Mallakpour S., Javadpour M. (2018). Sonochemical
assisted synthesis and characterization
of magnetic PET/Fe_3_O_4_, CA, AS nanocomposites:
Morphology and physiochemical properties. Ultrason
Sonochem..

[46] Vinosha P. A., Mely L. A., Jeronsia J. E., Krishnan S., Das S. J. (2017). Synthesis
and properties of spinel ZnFe_2_O_4_ nanoparticles
by facile co-precipitation route. Optik (Stuttg)..

[47] Brabers V. A. M. (1969). Infrared
Spectra of Cubic and Tetragonal Manganese Ferrites. physica status solidi (b)..

[48] Laurent S., Forge D., Port M., Roch A., Robic C., Vander Elst L., Muller R. N. (2008). Magnetic iron oxide nanoparticles:
synthesis, stabilization, vectorization, physicochemical characterizations,
and biological applications. Chemical reviews..

[49] Coromelci C., Neamtu M., Ignat M., Samoila P., Zaltariov M. F., Palamaru M. (2022). Ultrasound assisted synthesis of heterostructured TiO2/ZnFe2O4
and TiO2/ZnFe1. 98La0. 02O4 systems as tunable photocatalysts for
efficient organic pollutants removal. Ceram.
Int..

[50] Cullity, B. D. ; Stock, S. R. Elements of X-ray Diffraction, 3rd ed.; Prentice-Hall: New York, 2001.

[51] Köseoǧlu Y., Baykal A., Toprak M. S., Gözüak F., Basaran A. C., Aktas B. (2008). Synthesis and characterization of
ZnFe_2_O_4_ magnetic nanoparticles via a PEG-assisted
route. J. Alloys Compd..

[52] Andhare D. D., Jadhav S. A., Khedkar M. V., Somvanshi S. B., More S. D., Jadhav K. M. (2020). Structural and chemical
properties
of ZnFe_2_O_4_ nanoparticles synthesised by chemical
co-precipitation technique. Journal of Physics:
Conference Series.

[53] Suryanarayana, C. ; Norton, M. G. X-Ray Diffraction A Practical Approach. SpringerLink, 1998.

[54] Bindu P., Thomas S. (2014). Estimation of lattice strain in ZnO nanoparticles:
X-ray peak profile analysis. Journal of Theoretical
and Applied Physics..

[55] Yeap S. P., Lim J., Ooi B. S., Ahmad A. L. (2017). Agglomeration,
colloidal stability,
and magnetic separation of magnetic nanoparticles: collective influences
on environmental engineering applications. Journal
of Nanoparticle Research..

[56] Serantes D., Baldomir D. (2021). Nanoparticle size threshold for magnetic agglomeration
and associated hyperthermia performance. Nanomaterials..

[57] Bhattacharjee S. (2016). DLS and zeta
potential–what they are and what they are not?. Journal of controlled release..

[58] Park Y., Whitaker R. D., Nap R. J., Paulsen J. L., Mathiyazhagan V., Doerrer L. H., Song Y.-Q., Hurlimann M. D., Szleifer I., Wong J. Y. (2012). Stability of superparamagnetic
iron
oxide nanoparticles at different pH values: experimental and theoretical
analysis. Langmuir..

[59] Maguire C. M., Rösslein M., Wick P., Prina-Mello A. (2018). Characterisation
of particles in solution–a perspective on light scattering
and comparative technologies. Science and technology
of advanced materials..

[60] de
la Cruz E. F., Zheng Y., Torres E., Li W., Song W., Burugapalli K. (2012). Zeta Potential of Modified Multi-walled
Carbon Nanotubes in Presence of poly (vinyl alcohol) Hydrogel. Int. J. Electrochem Sci..

[61] Wang P., Keller A. A. (2009). Natural and Engineered
Nano and Colloidal Transport:
Role of Zeta Potential in Prediction of Particle Deposition. Langmuir..

[62] Mikelashvili V., Kekutia S., Markhulia J., Saneblidze L., Maisuradze N., Kriechbaum M., Almásy L. (2023). Synthesis
and characterization of citric acid-modified iron oxide nanoparticles
prepared with electrohydraulic discharge treatment. Materials..

[63] Sood A., Arora V., Shah J., Kotnala R. K., Jain T. K. (2016). Ascorbic
acid-mediated synthesis and characterisation of iron oxide/gold core–shell
nanoparticles. Journal of Experimental Nanoscience.

[64] Islam K., Haque M., Kumar A., Hoq A., Hyder F., Hoque S. M. (2020). Manganese Ferrite Nanoparticles (MnFe_2_O_4_): Size Dependence for Hyperthermia and Negative/Positive
Contrast Enhancement in MRI. Nanomaterials..

[65] Ozel F., Karaagac O., Tokay E., Kockar F., Kockar H. (2019). A simple way
to synthesize tartaric acid, ascorbic acid and their mixture coated
superparamagnetic iron oxide nanoparticles with high saturation magnetisation
and high stability against oxidation: characterizations and their
biocompatibility studies. J. Magn. Magn. Mater..

[66] Maisha N., Coombs T., Lavik E. (2020). Development
of a sensitive assay
to screen nanoparticles in vitro for complement activation. ACS biomaterials science & engineering..

[67] Sadat S. M., Jahan S. T., Haddadi A. (2016). Effects of
size and surface charge
of polymeric nanoparticles on in vitro and in vivo applications. Journal of Biomaterials and Nanobiotechnology..

[68] Braim F. S., Razak N. N. A. N. A., Aziz A. A., Dheyab M. A., Ismael L. Q. (2023). Optimization
of ultrasonic-assisted approach for synthesizing a highly stable biocompatible
bismuth-coated iron oxide nanoparticles using a face-centered central
composite design. Ultrasonics sonochemistry..

[69] Juvencio
Keijok W., Contreras Alvarez L.
A., Gomes A. M. d. S., Vasconcelos Campos F., Oliveira J. P. d., Guimaraes M. C. C. (2025). Optimized
Synthesis and Stabilization of Superparamagnetic Iron Oxide Nanoparticles
for Enhanced Biomolecule Adsorption. ACS Omega.

[70] Kowalczyk B., Lagzi I., Grzybowski B. A. (2011). Nanoseparations: Strategies for size
and/or shape-selective purification of nanoparticles. Curr. Opin. Colloid Interface Sci..

[71] Fouda M. F. R., Wahba M. A., El-Shahat M. F., ElKholy M. B., Mostafa S. A., Hussien A. I. (2013). Characterization
And Evaluation Of Nano-sized α-Fe_2_O_3_ pigments
synthesized Using Three Different Carboxylic
Acid. Adv. Mater. Lett..

[72] Segal B. G., Kaplan M., Fraenkel G. K. (1965). Measurement
of *g* Values in the Electron Spin Resonance Spectra
of Free Radicals. J. Chem. Phys..

[73] Alshahrani B., ElSaeedy H. I., fares S., Korna A. H., Yakout H. A., Maksoud M. I. A. A., Fahim R. A., Gobara M., Ashour A. H. (2021). The effect
of Ce^3+^ doping on structural, optical, ferromagnetic resonance,
and magnetic properties of ZnFe_2_O_4_ nanoparticles. Journal of Materials Science: Materials in Electronics.

[74] Dixit G., Pal Singh J., Srivastava R. C., Agrawal H. M. (2012). Magnetic resonance
study of Ce and Gd doped NiFe_2_O_4_ nanoparticles. J. Magn Magn Mater..

[75] Gharagozlou M. (2009). Synthesis,
characterization and influence of calcination temperature on magnetic
properties of nanocrystalline spinel Co-ferrite prepared by polymeric
precursor method. J. Alloys Compd..

[76] Mikhaylova M., Kim D. K., Bobrysheva N., Osmolowsky M., Semenov V., Tsakalakos T., Muhammed M. (2004). Superparamagnetism
of magnetite nanoparticles: dependence on surface modification. Langmuir..

[77] Baykal A., Deligöz H., Sozeri H., Durmus Z., Toprak M. S. (2012). Triethylene
Glycol Stabilized CoFe_2_O_4_ Nanoparticles. J. Supercond Nov Magn..

[78] Abbas M., Parvatheeswara Rao B., Kim C. (2014). Shape and size-controlled synthesis
of Ni Zn ferrite nanoparticles by two different routes. Mater. Chem. Phys..

[79] Tannous C., Gieraltowski J. (2008). The Stoner–Wohlfarth
model of ferromagnetism. Eur. J. Phys..

[80] Livesey K. L., Ruta S., Anderson N. R., Baldomir D., Chantrell R. W., Serantes D. (2018). Beyond the blocking model to fit nanoparticle ZFC/FC
magnetisation curves. Sci. Rep..

[81] Biederer S., Knopp T., Sattel T. F., Lüdtke-Buzug K., Gleich B., Weizenecker J. (2009). Magnetization response
spectroscopy of superparamagnetic nanoparticles for magnetic particle
imaging. J. Phys. D Appl. Phys..

[82] Ansari S. R., Imhoff E. D., Suárez-López Y. D. C., Melnyk A., Rinaldi-Ramos C. M., Teleki A. (2025). Flame-Made Doped Iron
Oxide Nanoparticles
as Tracers for Magnetic Particle Imaging. Chem.
Mater..

[83] Velazquez-Albino A. C., Imhoff E. D., Rinaldi-Ramos C. M. (2025). Advances
in engineering nanoparticles
for magnetic particle imaging (MPI). Sci. Adv..

[84] Salimi M., Wang W., Roux S., Laurent G., Bazzi R., Goodwill P., Liu G., Bulte J. W. M. (2025). MPI performance
of magnetic nanoparticles depends on matrix composition and temperature:
implications for in vivo MPI signal amplitude, spatial resolution,
and tracer quantification. Nanoscale Adv..

